# Transcriptome analysis of table grapes (*Vitis vinifera* L.) identified a gene network module associated with berry firmness

**DOI:** 10.1371/journal.pone.0237526

**Published:** 2020-08-17

**Authors:** Li Ma, Lingjun Sun, Yinshan Guo, Hong Lin, Zhendong Liu, Kun Li, Xiuwu Guo

**Affiliations:** 1 College of Horticulture, Shenyang Agricultural University, Shenyang, Liaoning, P.R. China; 2 Liaoning Institute of Pomology, Yingkou, Liaoning, P.R. China; Mediterranean Agronomic Institute of Chania, GREECE

## Abstract

Berry firmness is one of the main selection criteria for table grape breeding. However, the underlying genetic determinants and mechanisms involved in gene expression during berry development are still poorly understood. In this study, eighteen libraries sampled from *Vitis vinifera* L. cv. ‘Red Globe’ and ‘Muscat Hamburg’ at three developmental stages (preveraison, veraison and maturation) were analyzed by RNA sequencing (RNA-Seq). The firmness of ‘Red Globe’ was significantly higher than that of ‘Muscat Hamburg’ at the three developmental stages. In total, a set of 4,559 differentially expressed genes (DEGs) was identified between ‘Red Globe’ and ‘Muscat Hamburg’ in the preveraison (2,259), veraison (2030) and maturation stages (2682), including 302 transcription factors (TFs). Weighted gene coexpression network analysis (WGCNA) showed that 23 TFs were predicted to be highly correlated with fruit firmness and propectin content. In addition, the differential expression of the PE, PL, PG, β-GAL, GATL, WAK, XTH and EXP genes might be the reason for the differences in firmness between ‘Red Globe’ and ‘Muscat Hamburg’. The results will provide new information for analysis of grape berry firmness and softening.

## Introduction

Grape is a globally cultivated fruit, with 7.15 million hectares of area harvested and 79.18 million tons of production in 2018 (http://www.fao.org/faostat/en/#data/QC/visualize). Berry firmness is considered one of the most important traits for grape breeding. Grape firmness has been considered a measure of freshness [1], and the high berry firmness of table grape contributes to its desirable crunchy texture, which is an important factor that affects eating quality and prolongs postharvest shelf life with decreased loss during storage. Berry firmness is a typical quantitative trait attributed to polygenes. The berry flesh texture can be roughly divided into soft, medium, slightly firm and very firm based on firmness [2]. In recent years, a few studies on the identification of quantitative trait loci (QTLs) associated with firmness have been performed based on linkage maps and genome-wide association analyses, and the QTLs for firmness are located in different linkage groups (LGs), including 1, 3, 4, 5, 8, 9, 10, 13, 16 and 18 [2–6]. Although the QTL for firmness on LG 18 may be a promising locus and some candidate genes related to berry firmness regulation have been identified from this QTL region [3, 5–6], the gene function for berry firmness still needs to be validated in further studies.

During berry development and maturation, the total soluble solid (TSS) content increased and berry firmness decreased after veraison [7] and the berries exhibited obvious softening. Softening is a characteristic of ripening in most fleshy fruits, but excessive softening may lead to postharvest decay or consumer rejection [8]. In the process of fruit softening, the composition and structure of the cell wall change in a complex manner [9–10]. The synergistic action of cell wall-degrading enzymes or proteins manifests as pectin dissolution, neutral sugar loss, xylan depolymerization, cell wall relaxation and fruit hardness reduction [11–14]. Previous studies have shown that cell wall-related gene expression and multiple enzyme activities are involved in changes in berry firmness or softening, such as endo-1,4-β-glucanases, xyloglucan endo-transglycosylases (XTHs), β-galactosidases (β-Gals), and polygalacturonases (PGs) [9, 15–16], and cell wall swelling may be related to loosening of the xyloglucan–cellulose network and to pectin solubilization [10]. In grape, some genes involved in pectate lyase (PL) activity, xylosyltransferase activity, cellulose synthase activity, cation/calcium exchange and calcium binding have been proposed to be associated with berry firmness [2, 5, 17]. In addition, Wong et al. [17] found that the transcription factor (TF) APETALA2/Ethylene Responsive Factor (AP2/ERF) is associated with the presence of 1,4-beta-mannan endohydrolase, PLs, and PG, which are involved in berry firmness via regulation of cell wall degradation. These previous studies indicated that berry firmness involves complex multigene control. However, the genetic determinants and mechanisms involved in gene expression between hard and soft grape cultivars is still poorly understood.

In the present study, to expand the existing knowledge regarding the firmness of table grapes, transcriptome analysis was performed using the Illumina RNA-Seq method to clarify the transcriptome differences related with firmness between ‘Red Globe’ and ‘Muscat Hamburg’ during berry development.

## Materials and methods

### Plant material

Two table grape varieties (*Vitis vinifera* L.), namely, cv. ‘Red Globe’ and cv. ‘Muscat Hamburg’, grafted onto ‘Beta’ (*Vitis riparia* × *Vitis labrusca*) rootstock and grown at Shenyang Agricultural University (Liaoning, China; 41° 50’ N latitude, 123° 24’ E longitude, and elevation of 55 m) were used. ‘Muscat Hamburg’ is a widely planted variety worldwide with a strong Muscat aroma and soft pulp, and ‘Red Globe’ has a harder pulp than ‘Muscat Hamburg’ at the maturation stage. The grapevines were managed with the same methods as those used in commercial orchards. The berries of ‘Muscat Hamburg’ and ‘Red Globe’ have almost the same development period in Liaoning District. Five clusters of both cultivars were randomly collected at 56 days after full bloom (DAFB; one week before veraison, preveraison), 63 DAFB (veraison), 70 DAFB, 84 DAFB and 98 DAFB (maturation stage; commercial harvest time). A portion of each grape cluster was immediately frozen in liquid nitrogen after seed removal and stored at -80°C until analysis. Additional berries were used directly for TSS, total acid (TA), propectin, water-soluble pectin (WSP) and firmness analyses.

### TSS, TA and firmness analyses

For TSS estimation, pooled grape juice samples from 5 berries per cluster with five clusters per variety were prepared. The TSS content was analyzed for Bix using a digital pocket refractometer (PL-1, ATAGO, Japan), and the TA content was detected using an Agilent 1100 HPLC (Agilent Technologies, Palo Alto, CA) with a DAD detector and an Agilent SB-AQ column (4.6×250 mm, 5 μm) according to a previous method [18]. Firmness was determined for 25–30 berries (five to six berries per cluster) from five clusters using a TA.XT Plus Texture Analyzer (Stable Micro Systems, Surrey, UK) with a 2-mm diameter penetration probe (needle P/2). The penetration test of the whole grape berry (with skin) was performed at 1 mm/s for 6 mm after contacting the surface of the confection cube, and the results are expressed in N [19].

### Determination of propectin and WSP concentrations

The propectin and soluble pectin were quantified using the carbazole colorimetry method based on a previous study [8] with the modifications described below. One gram berry pulp mixed with 25 mL 95% ethylalcohol (v/v) after homogenization was used for propectin and water-soluble pectin (WSP) extraction, and 0.2 milliliter 1.5g/L carbazole ethanol solution was added to the final extracted solution and incubated in the dark for 30 min. The absorbance at 530 nm was measured by a UV-Vis spectrophotometer (UV-2600, Shimadzu, Japan), and different pectin contents were expressed as the mass percentage of the galacturonic acid produced.

### RNA extraction, preparation of RNA-Seq samples and RT-qPCR verification

**Fig 1:**
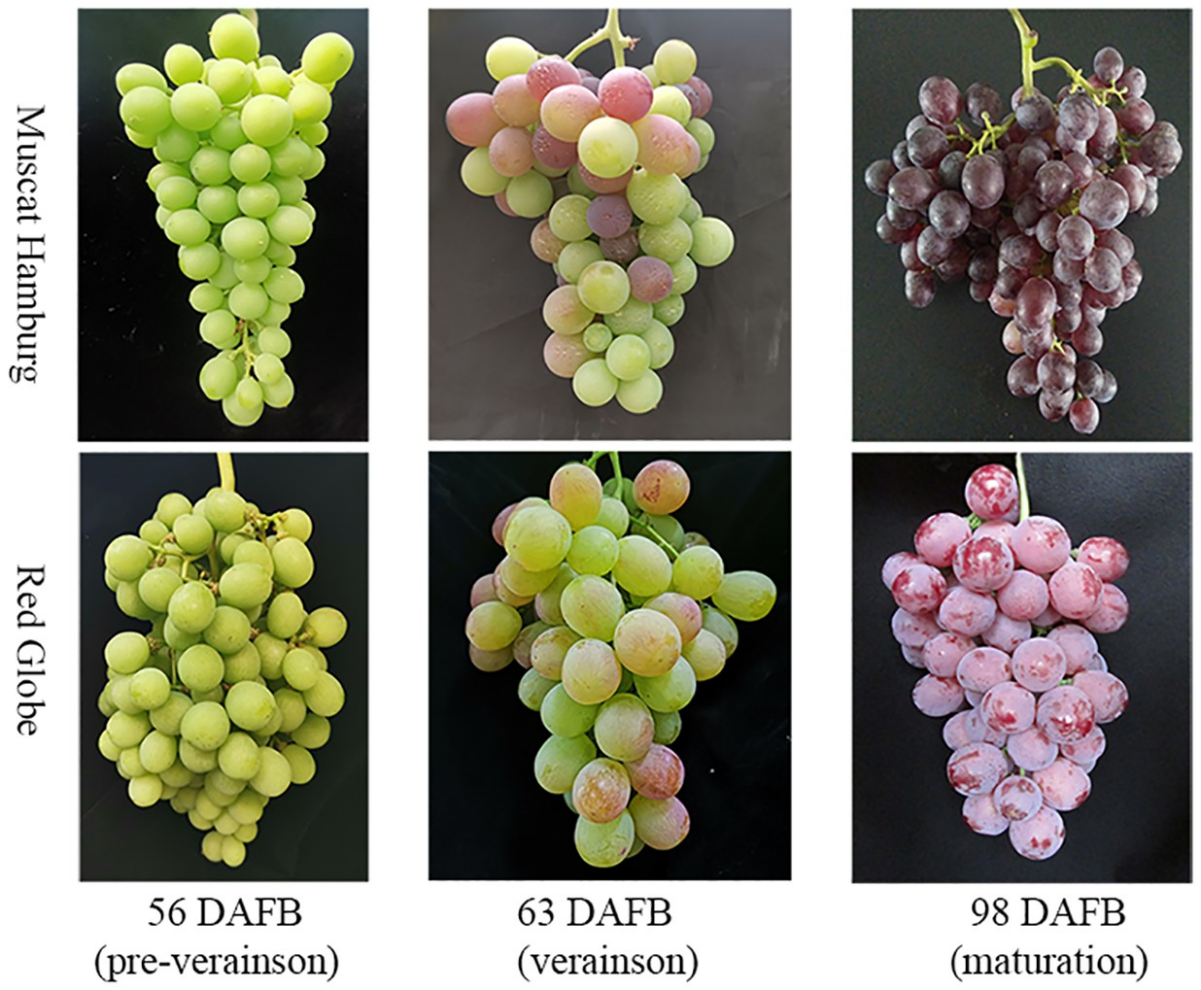
Images showing cluster-level changes in berries of ‘Red Globe’ and ‘Muscat Hamburg’ at the preveraison, veraison and maturation stages.

‘Muscat Hamburg’ and ‘Red Globe’ at three developmental stages (preveraison, veraison and maturation) with three biological replicates were used for RNA-Seq analysis (Fig 1). Total RNA was extracted using the Plant Total RNA Isolation Kit (SK8631, Sangon Biotech, Shanghai, China). The integrity of the RNA was examined using an Agilent 2100 Bioanalyzer (Agilent Technologies, Palo Alto, USA). cDNA synthesis was performed using the PrimeScript^™^ RT Kit (TaKaRa, Kusatsu, Japan) according to the manufacturer’s instructions. Three replications of samples at each developmental stage were used to construct eighteen cDNA transcriptome libraries and sequenced at Beijing Biomarker Technologies Co. Ltd. (Beijing, China). Sequencing libraries were generated using NEBNext UltraTM RNA Library Prep Kit for Illumina (NEB, USA) following manufacturer’s recommendations and index codes were added to attribute sequences to each sample. And then each library was sequenced using an Illumina NovaSeq 6000 (Illumina, San Diego, CA) in 150bp paired-end format according to the Illumina Paired-End Sequencing protocol. The average size of inserts in the library was 300 bp and ultimately generating a total of 0.83 billion clean reads.

RT-qPCR validation was performed with an ABI QuantStudio 6 Flex System (Applied Biosystems, Foster City, CA, USA). The reaction mixture was prepared using SYBR^®^ PremixExTaq^™^ II (Takara, Madison, WI, USA). The RT-qPCR primers used in this study were designed by Primer 3.0 (S1 Table). The expression level was calculated as 2^-ΔΔCt^ and normalized to the Ct value of *VvActin* [20].

### Analysis of the RNA-Seq data

The raw data of FASTQ format were firstly processed through in-house perl scripts. Clean data (clean reads) were obtained by removing reads containing adapters, reads with lengths below 50bp low-quality reads with poly-N >10% from the raw data, and the Q30, GC content and sequence duplication level of the clean data were calculated. Then, the clean reads were mapped to the grape reference genome (GCA_000003745.2) [21] for further analysis using TopHat2 (2.0.13) [22] with the parameters of–library-type ‘fr-unstranded’–splice-mismatches ‘0’–min-intron-length ‘50’–max-intron-length ‘50000’. Gene expression levels were normalised by determining the fragments per kilobase of transcript per million fragments mapped (FPKM). The differentially expressed gene (DEG) analysis was performed on the raw count data using the DESeq R package (1.10.1). Genes with |Fold Change| ≥ 2 and false discovery rate (FDR) < 0.01 were regarded as DEGs.

To further investigate the functional associations of the DEGs, we performed Kyoto Encyclopedia of Genes and Genomes (KEGG) pathway analysis by using the public database (https://www.kegg.jp/kegg/). KOBAS (2.0) software [23] (http://kobas.cbi.pku.edu.cn/help.do) with -num_descriptions 100 -num_alignments 100 -evalue 1e-5 was used to test the statistical enrichment of differential expression genes in KEGG pathways. Pathways with Pvalue ≤ 0.05 were defined as genes that display significant levels of differential expression. TF identification and classification were conducted by using the DEGs sequence as input against transcription factor database iTAK(1.6) [24] (http://itak.feilab.net/cgi-bin/itak/index.cgi,version 18.12) based on 197 plant genome information. The RNA-Seq data were deposited in the Sequence Read Archive (SRA) at NCBI under accession number PRJNA635268.

### WGCNA

Weighted gene coexpression network analysis (WGCNA) was conducted using R with the WGCNA (1.42) package [25]. The 4,559 DEGs with FPKM values > 0.5 were used for coexpression network analysis. The modules were obtained using the automatic network construction function “blockwise” by using default settings, except that the soft power was 18, minModuleSize = 5 and cutHeight = 0.25. The eigengene value was calculated for each module and used to test the association with firmness and propectin. The total connectivity and intramodular connectivity were calculated with weighted and Pearson correlations function.

### Statistical analysis

The data were obtained as the means ± standard deviation (SD) from biological replicates, Differences between groups were assessed using unpaired Student’s t test at P ≤ 0.05 using SPSS Statistics 21.0 software (IBM, Armonk, NY, USA). Principal component analysis (PCA) was conducted using the ‘prcomp’ function in the R package ‘stats’.

## Results

### Phenotypic characterization

During berry development, the TSS content increased and TA content decreased in both ‘Muscat Hamburg’ and ‘Red Globe’ from preveraison (56 DAFB) to maturation (98 DAFB). However, the TSS content in ‘Muscat Hamburg’ was higher than that in ‘Red Globe’ at the same developmental stage. The TA content in ‘Red Globe’ was lower than that in ‘Muscat Hamburg’ from 56 DAFB to 84 DAFB, but there was no significant difference between these two cultivars at pre-veraison and maturation. The pulp firmness of ‘Red Globe’ was significantly higher than that in ‘Muscat Hamburg’ from preveraison to maturation, especially in preveraison and veraison (Table 1).

**Table 1:** Variations of pulp firmness, total soluble solid and total acid in ‘Red Globe’ and ‘Muscat Hamburg’.

DAFB^a^	Pulp firmness (*N*)		Total Soluble Solid (%)		Total acid (g/100g FW)	
	‘Red Globe’	‘Muscat Hamburg’	‘Red Globe’	‘Muscat Hamburg’	‘Red Globe’	‘Muscat Hamburg’
56 (Pre-veraison)	2.88 ± 0.03*	1.97 ± 0.23*	3.75 ± 0.14*	6.50 ± 0*	1.14 ± 0.03	1.22 ± 0.07
63 (Veraison)	2.74 ± 0.14*	1.71 ± 0.03*	6.75 ± 0.14*	8.50 ± 0*	0.51 ± 0.01*	0.93 ± 0.03*
70	1.41 ± 0.15*	0.64 ± 0.16*	7.50 ± 0*	12.25 ± 0.14*	0.49 ± 0.01*	0.82 ± 0.11*
84	0.68 ± 0.1*	0.31 ± 0.02*	14.50 ± 0*	18.00 ± 0.29*	0.37 ± 0.02*	0.53 ± 0.03*
98 (Maturation)	0.44 ± 0.06*	0.26 ± 0.08*	17.50 ± 0*	18.75 ± 0.14*	0.39 ± 0.01	0.41 ± 0.02

^a^ Days Post Anthesis

* Statistically significant differences(Student’s t test, p≤0.05) between ‘Red Globe’and ‘Muscat Hamburg’ at the same evaluation moment considering a complete set of sampling data for each parameter. Data are presented as the means ± standard deviation (SD)

### Transcriptomic profiling of berry development in ‘Red Globe’ and ‘Muscat Hamburg’

Eighteen mRNA samples from two varieties at preveraison, veraison and maturation (Fig 1) were sequenced, and approximately 0.83 billion clean reads were obtained after removing adapter sequences and reads of low quality and those with more than 10% N bases from the raw reads. The average number of clean reads per sample was about 46.16 million. The Q30 percentages of the clean data for all samples were higher than 92.11%, and the GC contents of the clean data for all samples ranged from 46.48 to 48.81%. For further analysis, the high-quality clean reads were mapped to the grape reference genome using TopHat2, and total 690.4 million uniquely mapped reads of all samples were used for read counting (S2 Table).

**Fig 2:**
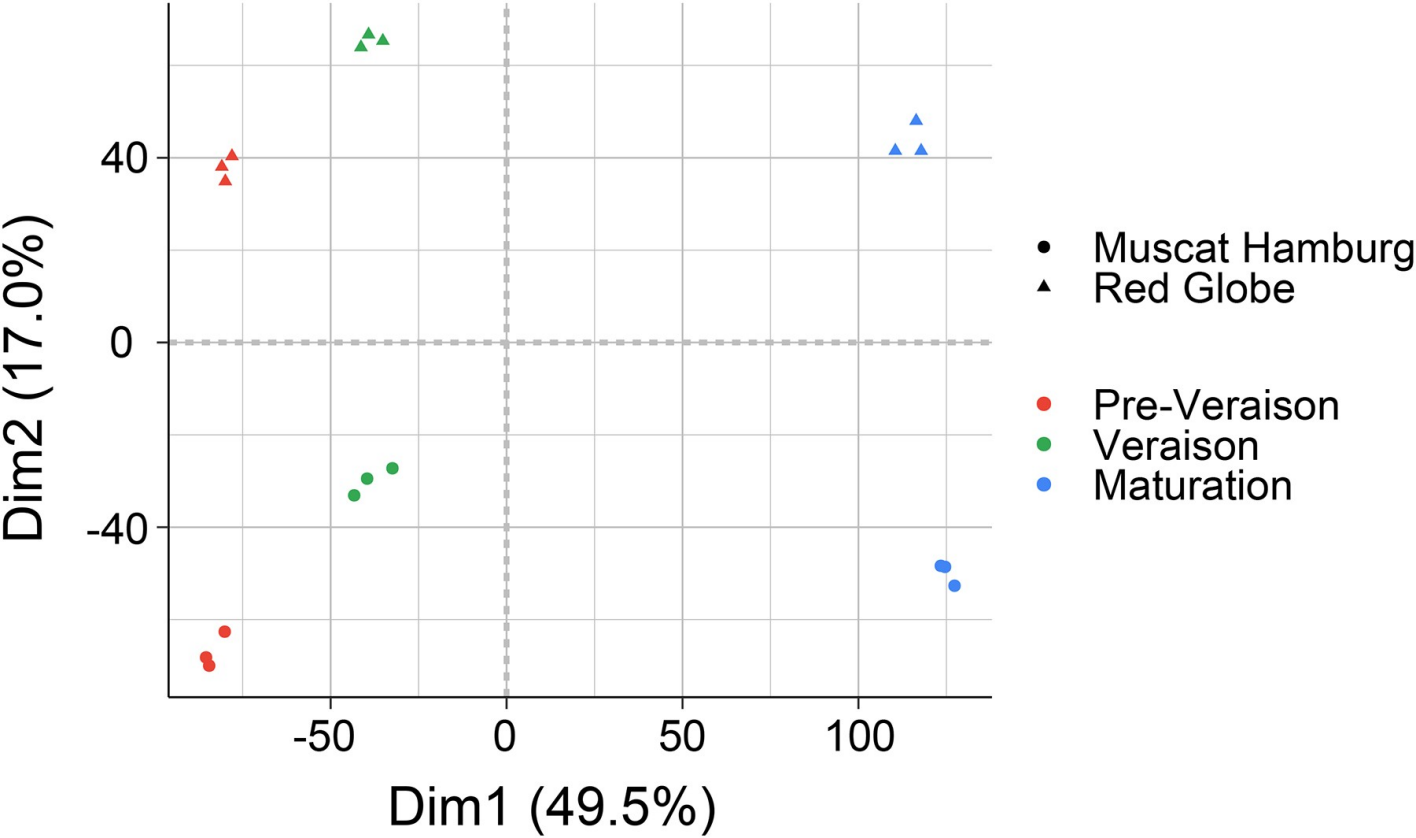
Principal component analysis of the RNA-Seq data. *Circle* and *Triangle* represent Muscat Hamburg and the Red Globe, respectively. The three different colors represent different development stages.

The principal component analysis (PCA) of the RNA-Seq data revealed the heterogeneity of all samples in respect to the cultivar and developmental stage based on gene expression. The first principal component (Dim1) contributed 49.5% of the variance and clearly discriminated developmental stages. Dim2 described 17% of the variance and discriminated the cultivars. Dim1 and Dim2 accounted for 62.9% of the principal components, and PCA revealed no differences between the three replicates at each developmental stage of two varieties (Fig 2).

**Fig 3:**
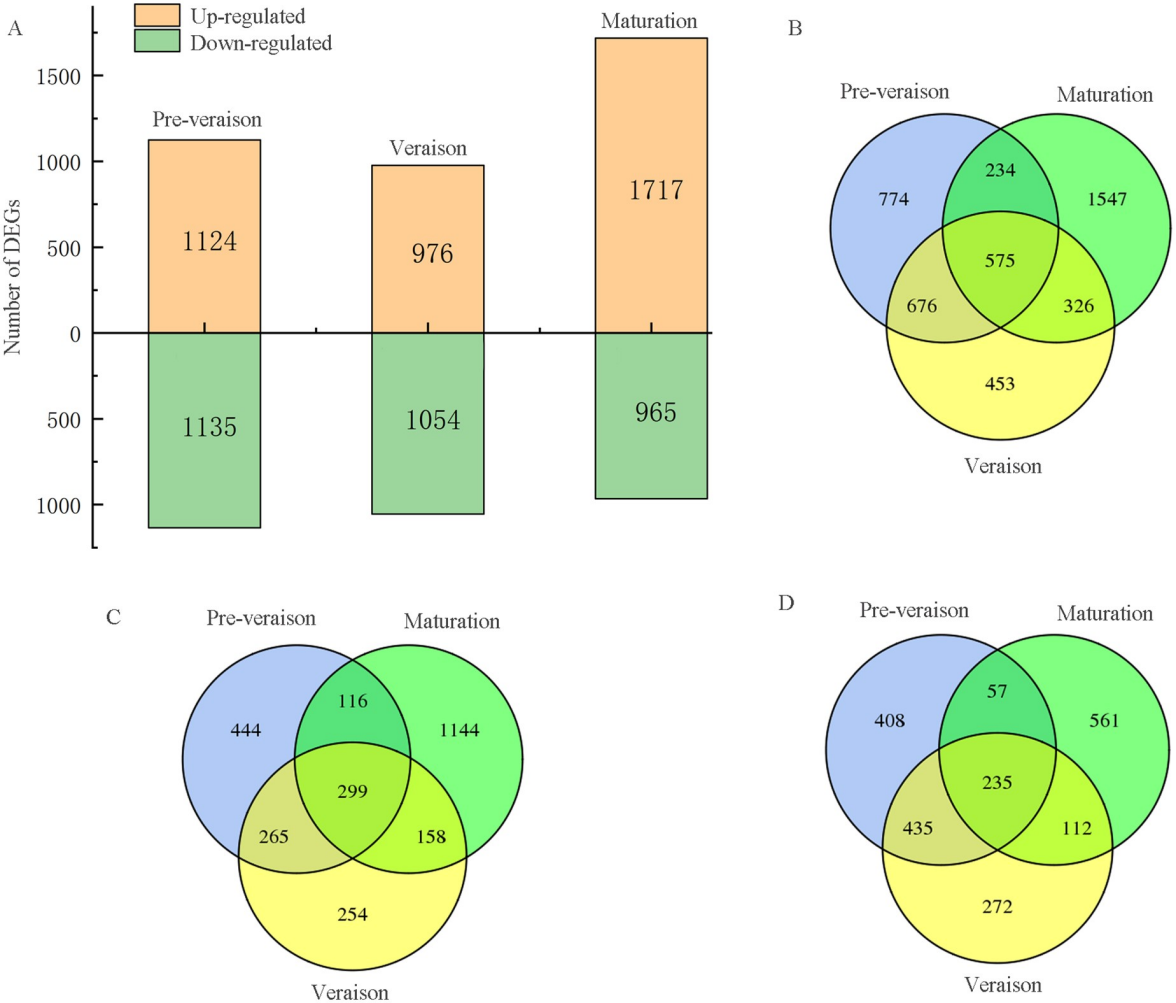
Analysis of DEGs between ‘Red Globe’ and ‘Muscat Hamburg’ at three developmental stages. (A) Number of up- and downregulated DEGs, using log2 fold change (|FC|) ≥ 2. (B) Venn diagram of unique and common DEGs between ‘Red Globe’ and ‘Muscat Hamburg’ at the preveraison, veraison and maturation stages. (C) Venn diagram of upregulated DEGs between ‘Red Globe’ and ‘Muscat Hamburg’ at the preveraison, veraison and maturation stages. (D) Venn diagram of downregulated DEGs between ‘Red Globe’ and ‘Muscat Hamburg’ at the preveraison, veraison and maturation stages.

In this study, a total of 4,559 DEGs with fold changes greater than or equal to 2 were identified at three developmental stages (S3 Table). Of these DEGs, 2,259 (1,124 upregulated, 1,135 downregulated), 2030 (976 upregulated, 1,054 downregulated) and 2682 (1,717 upregulated, 9,65 downregulated) were identified in ‘Red Globe’ compared to ‘Muscat Hamburg’ in the preveraison, veraison and maturation stages, respectively (Fig 3A). In addition, 575 DEGs were common up- or downregulated genes between ‘Muscat Hamburg’ and ‘Red Globe’ in the three stages (preveraison, veraison and maturation), and 2,774 DEGs were unique up- or downregulated genes between ‘Muscat Hamburg’ and ‘Red Globe’ in the preveraison, veraison or maturation stage (Fig 3B). A total of 299 DEGs were commonly upregulated between ‘Muscat Hamburg’ and ‘Red Globe’ in the three stages (Fig 3C), and 235 DEGs were commonly downregulated between ‘Muscat Hamburg’ and ‘Red Globe’ in the three stages (Fig 3D).

**Fig 4:**
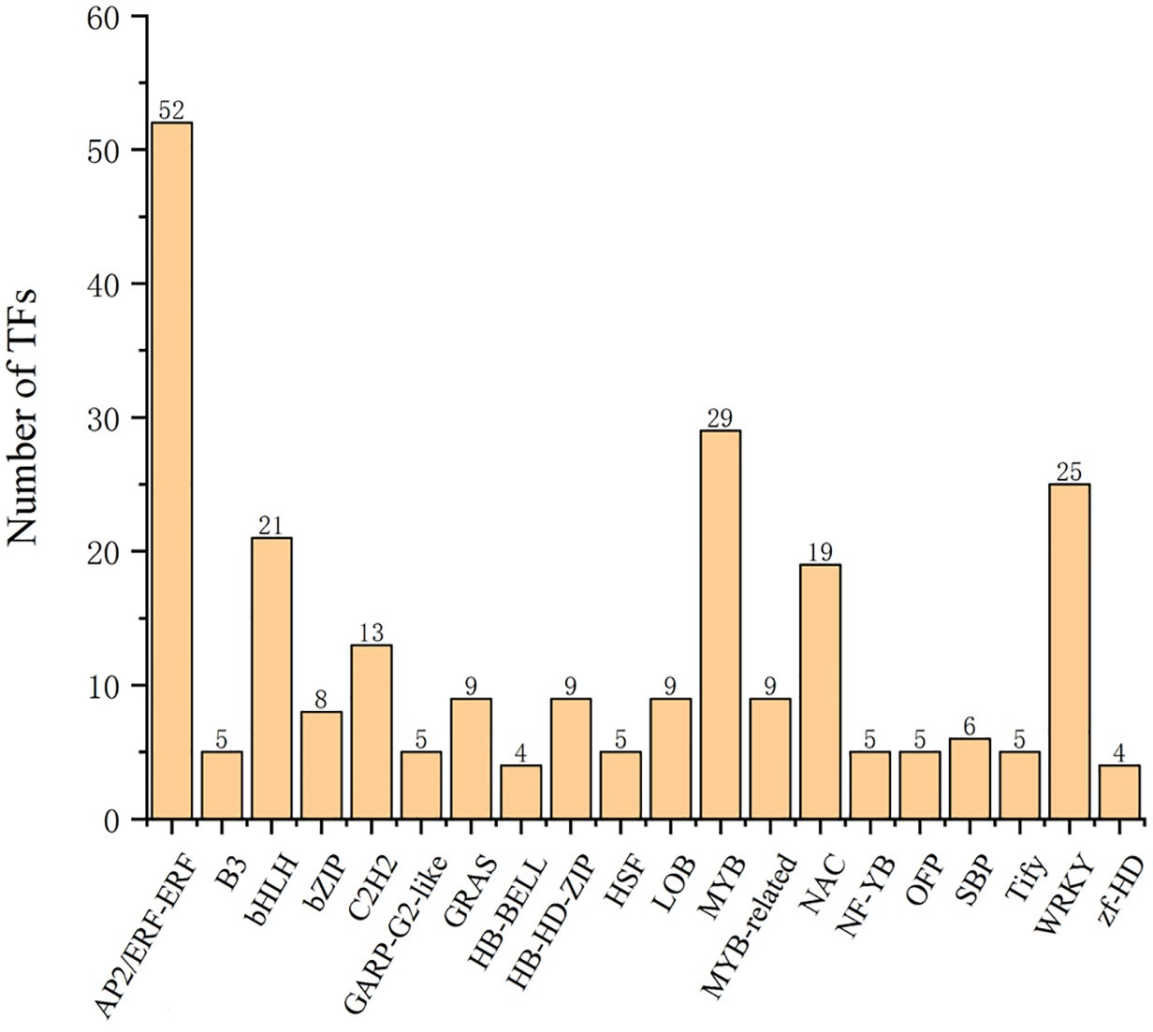
Enrichment analysis of DEGs. Top 20 pathways enriched in KEGG. The X-axis indicates the rich factor, and the Y-axis indicates the KEGG pathway. The larger the rich factor is, the higher the degree of enrichment. Q-value range, 0 to 1; the closer to zero the value is, the more significant the enrichment.

Functional and pathway assignments of the 4,559 DEGs with KEGG classification revealed that the plant-pathogen interaction signaling pathway, phenylpropanoid pathway and flavonoid pathway were the top 3 pathways with the greatest number of DEGs. In addition, the galactose metabolism pathway and pentose and glucuronate interconversion pathways, which may regulate cell wall metabolism and remodeling, were included among the top 20 enriched pathways (Fig 4, S3 Table).

**Fig 5:**
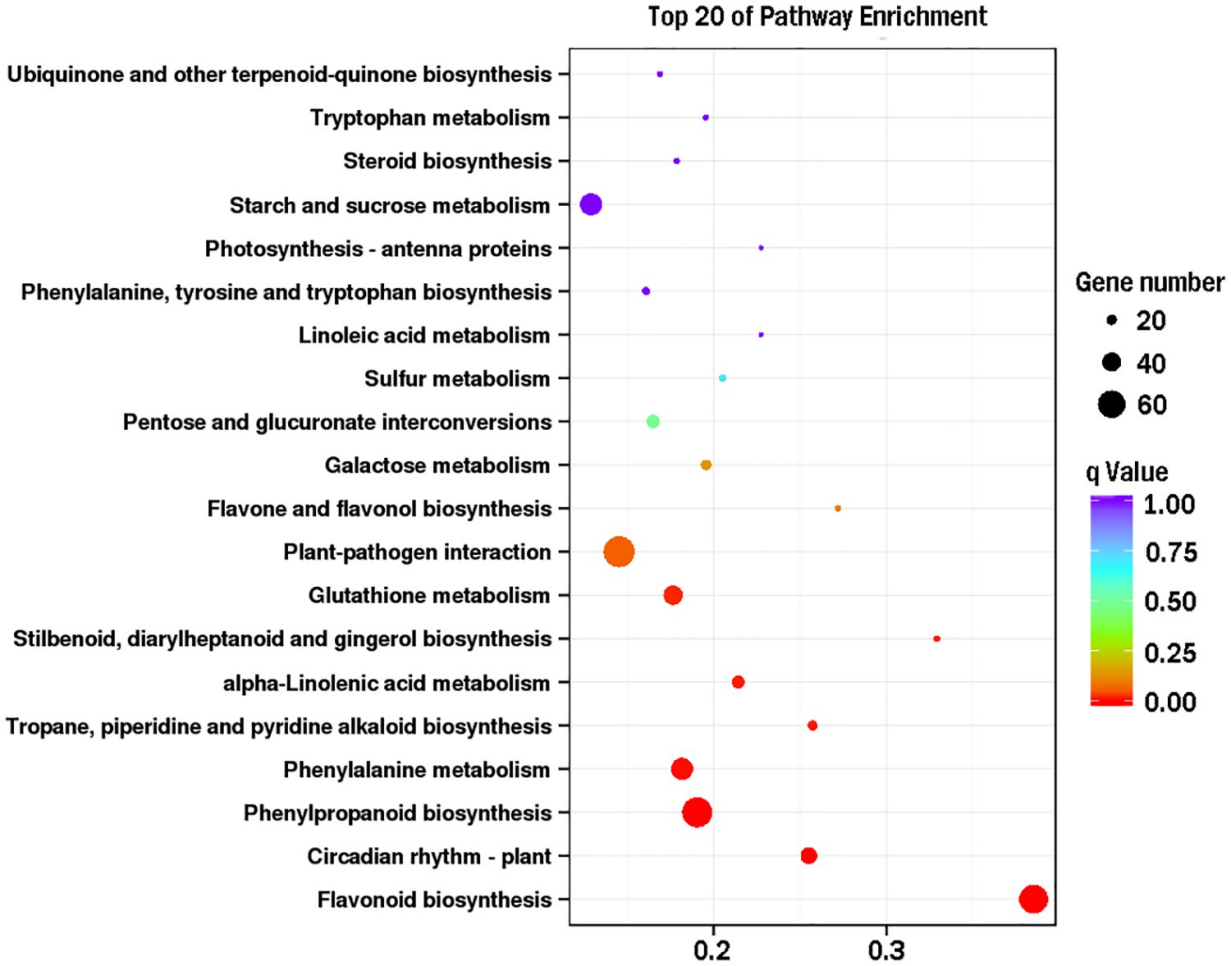
Relative abundance and distribution of the top 20 TF families between ‘Red Globe’ and ‘Muscat Hamburg’ at three developmental stages among the DEGs.

A total of 302 TF unigenes representing 47 TF families were predicted from 4,559 DEGs between ‘Muscat Hamburg’ and ‘Red Globe’ in the three sampling stages (S4 Table). The top 20 TF families are listed in Fig 5 The 5 largest groups among these families were the AP2/ERF-ERF (17.2%), MYB (9.6%), WRKY (8.3%), bHLH (7.0%), and NAC (6.3%) families (Fig 5), representing more than half (52.6%) of all TFs.

TFs have a potential effect on berry firmness, and pectin is an important component of cell wall synthesis and remodeling. Within the set of DEGs, we focused on the portions of the grape transcriptome related to TF and pectin compound metabolism.

### Identification of firmness- and propectin-associated genes by coexpression network analysis

**Fig 6:**
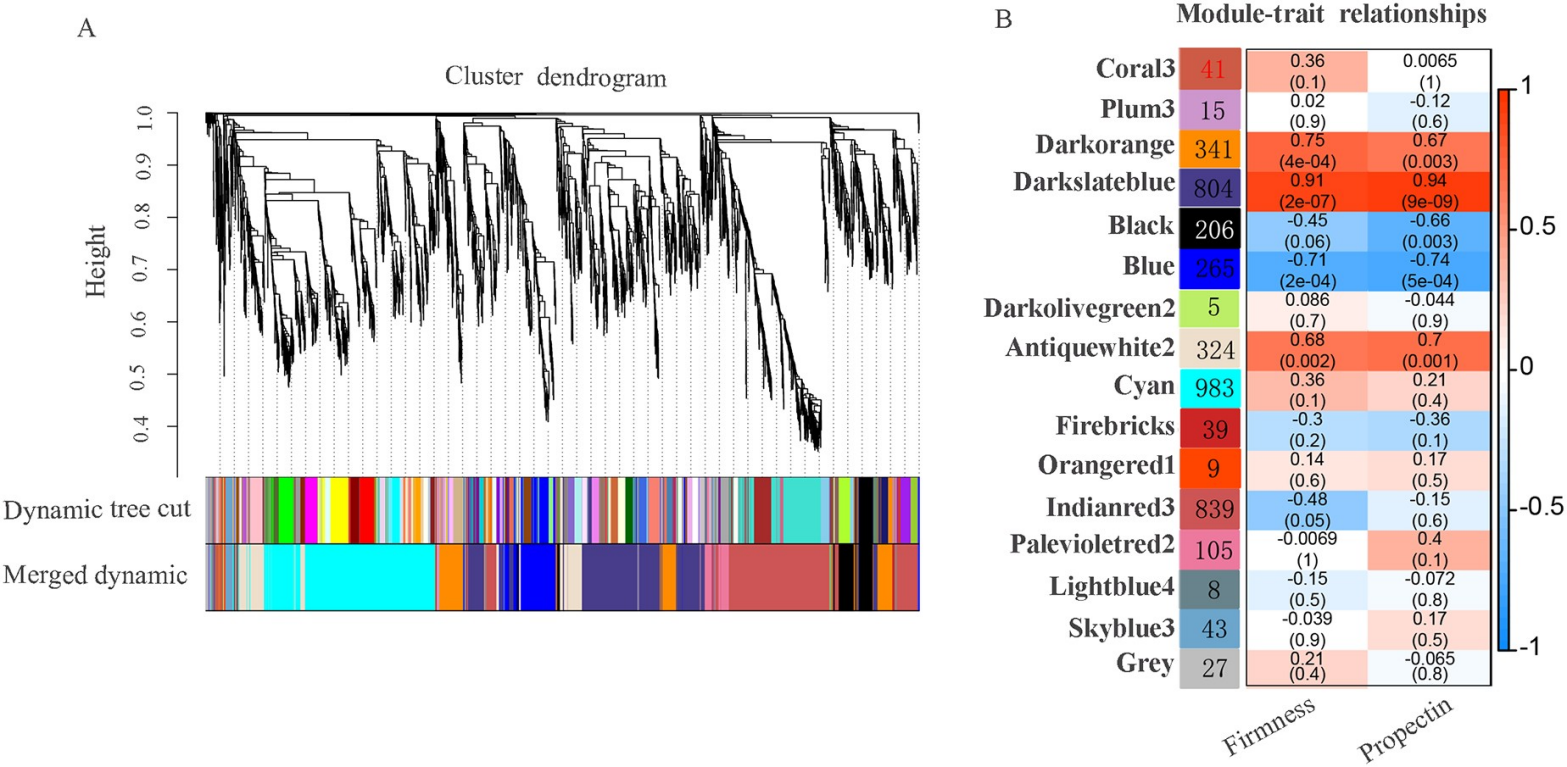
Coexpression network analysis of DEGs between ‘Red Globe’ and ‘Muscat Hamburg’ at three developmental stages. (A) Hierarchical cluster tree showing 16 modules of coexpressed genes. Each of the 4,559 DEGs is represented by a leaf in the tree, and each of the sixteen modules is represented by a major tree branch. (B) Heatmap of correlations and corresponding p-values for 16 gene modules. The left panel shows 16 modules and the number of genes in each module. The upper number shows the correlation from −1 to 1, and the lower number shows the p-value in the right panel.

The WGCNA showed that the DEGs comprised sixteen coexpression modules (Fig 6A). Most of the DEGs fell into the modules ‘Cyan’, ‘Indianred3’ and ‘Darkslateblue’. The module ‘Cyan’, with 983 DEGs, contained the greatest number of genes, followed by the modules ‘Indianred3’ and ‘Darkslateblue’, which featured 839 and 804 DEGs, respectively. The ‘Darkolivegreen2’ module was the smallest, with only 5 DEGs. The module ‘Grey’ was used for unassigned genes and did not represent a real module (Fig 6B, S5 Table).

The DEGs in the coexpression modules showed that a significant correlation with firmness, with the coefficient varying from -0.71 to 0.91, and that for the relationship between the DEGs and the protopectin content varied from -0.74 to 0.94. In particular, the module ‘Darkslateblue’ showed a significant positive correlation (p < 0.001) with firmness (r = 0.91) and propectin (r = 0.94) in berries, and the module ‘Blue’ showed a negative correlation (p < 0.001) with firmness (r = 0.71) and propectin (r = 0.74) in berries. In addition, firmness was highly positively correlated with gene expression in the ‘Darkorange’ module, with a coefficient of 0.75 (p < 0.001). The ‘Darkslateblue’, ‘Blue’ and ‘Darkorange’ modules were composed of 1410 genes, among which the ‘Darkslateblue’ module, as the largest module, included 804 DEGs. The ‘Darkorange’ module contained 341 DEGs, while the ‘Blue’ module had 265 DEGs (Fig 6B, S5 Table).

### Genes related to pectin metabolism during berry development

**Fig 7:**
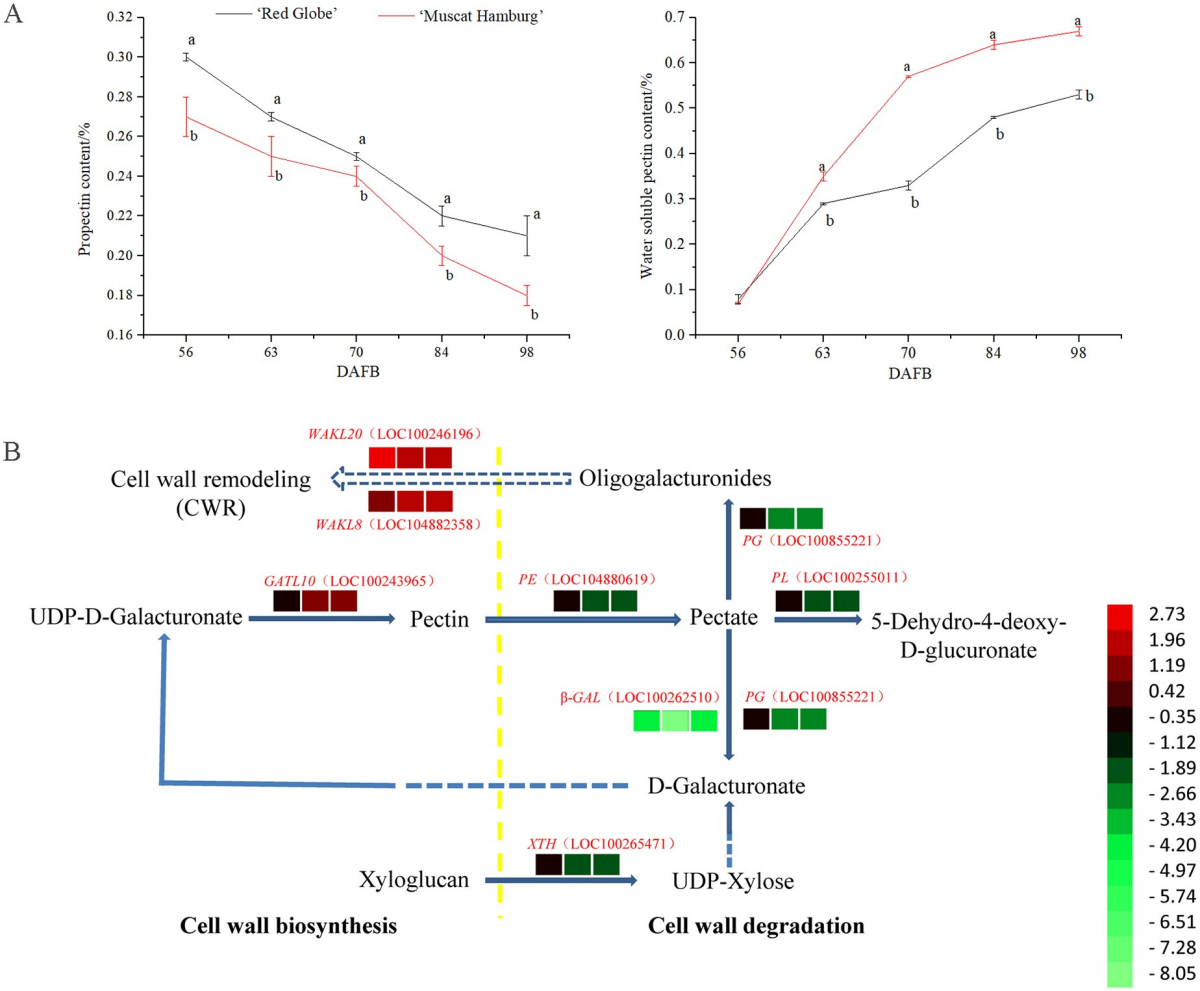
Changes in physiological and metabolic characteristics related to pectin during berry development. (A) Changes in the levels of propectin and water-soluble pectin. (B) Differential expression of genes involved in the pectin metabolic pathway between ‘Red Globe’ and ‘Muscat Hamburg’. The red-to-green scale indicates upregulation (red) or downregulation (green).

In this study, the propectin content decreased, and water-soluble pectin (WSP) accumulated in both ‘Muscat Hamburg’ and ‘Red Globe’ during berry development (Fig 7A). The propectin content in ‘Red Globe’ was higher than that in ‘Muscat Hamburg’ from 56 DAFB to 105 DAFB, even though the propectin content decreased in both ‘Muscat Hamburg’ and ‘Red Globe’. There was no significant difference in WSP content between ‘Red Globe’ and ‘Muscat Hamburg’ at 56 DAFB (preveraison). However, the WSP content increased dramatically in ‘Muscat Hamburg’ after veraison (63 DAFB), and the WSP content in ‘Muscat Hamburg’ was higher than that in ‘Red Globe’ from 63 DAFB to 105 DAFB (Fig 7A).

In the ‘Blue’, ‘Darkslateblue’ and ‘Darkorange’ modules, we found twenty-three TFs significantly associated with changes in firmness and propectin content, such as bHLH (LOC100855101, LOC100243748), AP2/ERF-ERF (LOC100253072) and NAC (LOC100252516) (S6 Table). In addition, three transcripts, encoding a pectinesterase (PE, LOC104880619), PG (LOC100855221) and PL (LOC100255011), were assigned to the module ‘Blue’. Three transcripts, encoding wall-associated receptor kinase-like (WAKL, LOC100246196), galacturonosyltransferase-like (GATL, LOC100243965) and xyloglucan endo-transglycosylase (XTH, LOC100265471), were assigned to the module ‘Darkslateblue’. Two transcripts, encoding WAKL (LOC104882358) and β-galactosidase (β-GAL, LOC100262510), were assigned to the module ‘Darkorange’.

The expression of PE, PL, PG and β-GAL, which are involved in pectin metabolism, was significantly downregulated in ‘Red Globe’ after veraison. The gene GATL, which is involved in pectin biosynthesis, was upregulated in ‘Red Globe’ compared to ‘Muscat Hamburg’. In addition, the levels of two transcripts encoding WAKL, which is related to cell wall remodeling, were significantly increased in ‘Red Globe’, and the levels of transcripts encoding XTH were downregulated in ‘Red Globe’ (Fig 7B).

### RT-qPCR verification of gene expression

**Fig 8:**
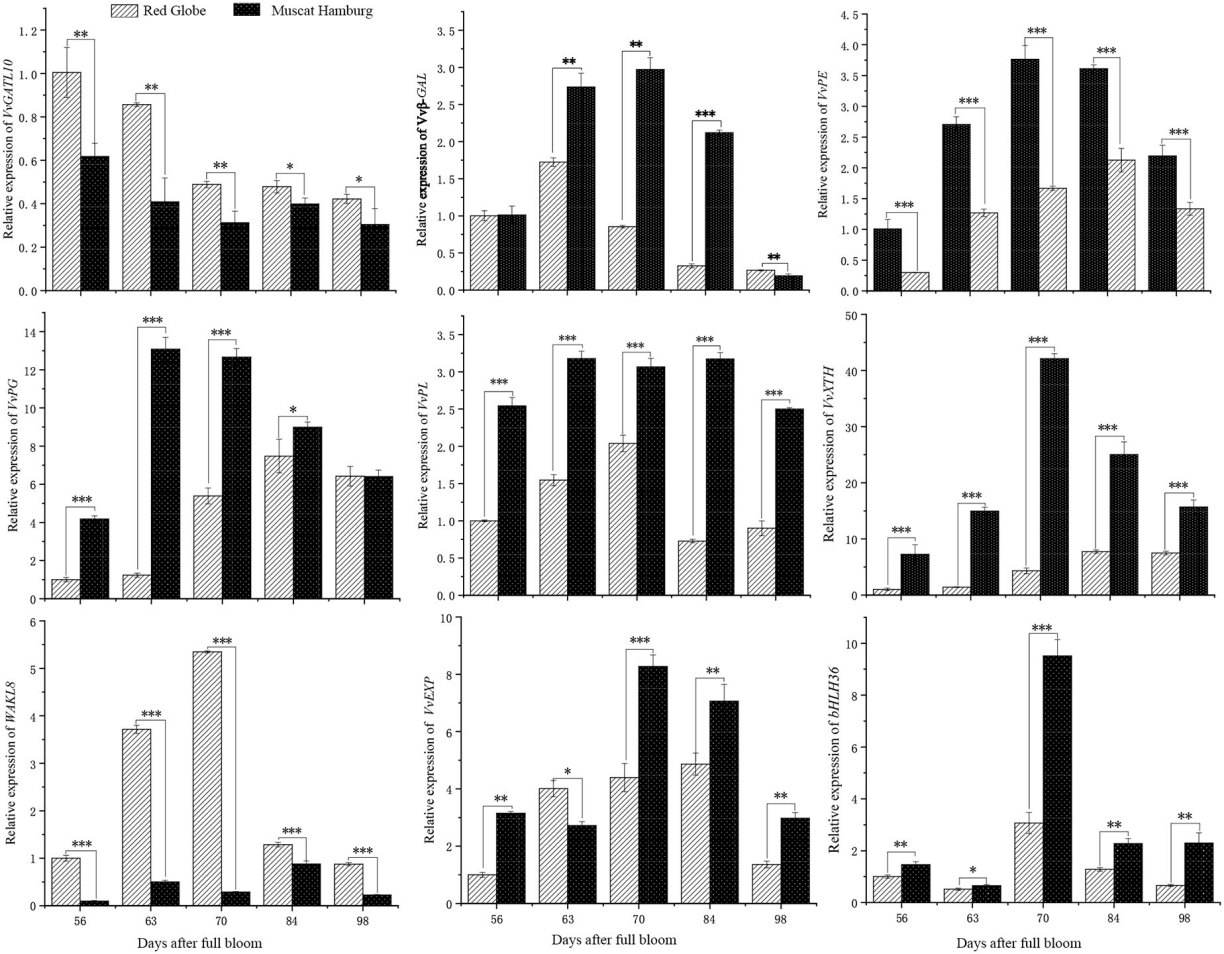
RT-qPCR validation of selected firmness-related DEGs between ‘Red Globe’ and ‘Muscat Hamburg’ during berry development. The x-axis represented different sampling date, while relative expression levels for the y-axis. Data represented the mean of three biological replicates. Error bars represented standard deviations (SD). P values were calculated by Student’s t test. *P < 0.05, **P < 0.01,***P < 0.001.

To further validate the RNA-Seq data, we examined the expression of a set of 9 firmness-related candidate genes by RT-qPCR. As shown in Fig 8, these genes included TF, cell wall degradation and remodeling-associated genes. The expression of *VvGATL10* (LOC100243965) was mainly downregulated in ‘Red Globe’ during ripening, and the expression level of this gene in ‘Red Globe’ was higher than that in ‘Muscat Hamburg’. The expression of *VvWAKL8* (LOC104882358) in ‘Red Globe’ was upregulated from 56 DAFB to 70 DAFB and then downregulated from 70 DAFB to 98 DAFB, and the gene *VvWAKL8* was significantly higher expressed in ‘Red Globe’ comparison with that in ‘Muscat Hamburg’. The expression levels of *VvXTH* (LOC100265471), *VvPE* (LOC104880619), *VvPG* (LOC100855221) and *VvPL* (LOC100255011) in ‘Red Globe’ were lower than those in ‘Muscat Hamburg’ during berry development. In addition, the expression levels of *Vvβ-GAL* (LOC100262510), *VvEXP* (LOC100260158) and *VvbHLH36* (LOC100243748) in ‘Red Globe’ were significantly downregulated in ‘Muscat Hamburg’ after veraison (63 DAFB), except that of *Vvβ-GAL* at 98 DAFB. The RT-qPCR data were consistent with the results obtained from transcriptome analysis.

## Discussion

For table grapes, mechanical properties are considered the most important factors that affect eating quality [26], and the firmness of the berry is considered to be a measure of its freshness [1]. In the present study, berry firmness decreased in both the hard-pulp cultivar ‘Red Globe’ and the soft-pulp cultivar ‘Muscat Hamburg’ from veraison to maturation, which is consistent with previous studies [7, 27]. On the other hand, the firmness of ‘Red Globe’ was higher than that of ‘Muscat Hamburg’ during the softening process. These results indicate that berry firmness is controlled by a complex multigene network.

The cell wall is a dynamic structure, and disintegration of the cell wall is the main reason for texture change and softening during berry maturation [15, 28]. This process involves concerted changes in cell wall-related gene expression and multiple enzyme activities. Pectins play an important role in cell-to-cell adhesion as the most structurally complex plant cell wall polysaccharides and are mainly divided into homogalacturonan (HG), rhamnogalacturonan 1 (RG-I) and rhamnogalacturonan II (RG-II). Following secretion of the methylated HG to the apoplast, pectin methylesterases (PMEs) hydrolyze the constituent methyl esters, yielding HG with a low degree of methylation that can then be cleaved by PL or by PG [29]. Previous studies have shown that PG participates in fruit firmness and softening by partially solubilizing the pectin fraction of the cell walls [30–31]. In this study, the expression of *VvPG* was upregulated at veraison and downregulated at maturation in ‘Muscat Hamburg’, and *VvPL* showed an expression trend similar to that of *VvPG*. The results show that the *VvPG1* and *VvPL* transcripts were positively correlated with berry softening [7, 32]. At the same time, it is notable that the expression of *VvPG* and *VvPL* in ‘Muscat Hamburg’ was higher than that in ‘Red Globe’. The results suggest that the difference in the expression of *VvPG* and *VvPL* may be involved in the difference in firmness between hard and soft berries.

Expansins (EXPs) exhibit no detectable hydrolase or transglycosylase activity toward cell wall-localized proteins [33], which disrupts noncovalent linkages at the cellulose–hemicellulose interface and perturbs the network [34]. Suppression of the Exp1 protein substantially inhibited tomato fruit softening [11]. In grape, previous studies have suggested that XTH regulates berry firmness [5, 17]. XTH may work together with EXP and PL to regulate berry softening [35–36]. In this study, *VvXTH* and *VvEXP* were highly expressed around veraison and then downregulated, similar to previous results [36]. However, *VvXTH* and *VvEXP* were significantly upregulated in ‘Muscat Hamburg’ compared with ‘Red Globe’. In addition, the transcript encoding *Vvβ-GAL* showed a similar gene expression pattern as that of *VvEXP*, suggesting that *Vvβ-GAL* may be related to berry firmness, as these genes are involved in fruit softening [37–39].

Signal transduction in the cell wall and intracellular signal transduction play a key role in plant development and environmental signal reception [40]. Wall-associated kinases (WAKs) are a family of proteins that can sense extracellular signals. They are transmembrane receptors that can directly bind to pectin residues (OGs) in the cell wall [41] and participate in the maintenance of cell integrity [40]. In this study, the transcript *VvWAKL8* was closely associated with berry firmness. The transcript was more highly expressed in ‘Red Globe’ than in ‘Muscat Hamburg’ during berry development. *Vv*WAKL was highest expressed at 70DAFB, and then downregulated until maturation. It is suggested that the *WAK* gene may be a key factor associated with high berry firmness. The high expression level of the *WAK* gene in hard berries may increase the chance of pectin residue binding with *WAK* and promote the maintenance of relatively high berry firmness.

Furthermore, some TFs have been found to regulate fruit ripening, which is characterized by softening in most fleshy fruits [42, 43]. TFs are sequence-specific DNA-binding proteins that interact with the cis-acting element in the promoter regions of corresponding target genes and modulate gene expression [44]. A total of 126 bHLH TFs have been identified and divided into 24 subfamilies in grape [45]. In this study, 21 *VvbHLHs* were differentially expressed between ‘Red Globe’ and ‘Muscat Hamburg’. RNA-Seq and RT-qPCR analysis showed that *VvbHLH36* was expressed at lower levels in ‘Red Globe’ than in ‘Muscat Hamburg’. In particular, *VvbHLH36* was significantly upregulated after veraison in ‘Muscat Hamburg’. These results suggest that *VvbHLH36* may be a potential regulator that works together with cell wall metabolism and remodeling genes to determine berry firmness.

## Conclusions

Functional analysis of the transcriptome during grape berry ripening of ‘Red Globe’ and ‘Muscat Hamburg’ allowed the identification of DEGs associated with the firmness of table grape flesh between hard and soft berries. These results suggest that the difference in the expression levels of genes related to cell wall metabolism-related enzymes and TFs may result in differences in firmness between ‘Red Globe’ and ‘Muscat Hamburg’.

## Supporting information

S1 Table Primers used for RT-qPCR validation. (DOCX)

S2 Table Statistical summary for sequence quality control and mapped data of the samples. (DOCX)

S3 Table Differentially expressed genes between ‘Red Globe’ and ‘Muscat Hamburg’ at the preveraison, veraison and maturation stages. (XLSX)

S4 Table Differentially expressed transcription factors between ‘Red Globe’ and ‘Muscat Hamburg’ at the preveraison, veraison and maturation stages. (XLSX)

S5 Table Differentially expressed genes were assigned to models. (XLSX)

S6 Table The transcription factors and cell wall metabolism-related genes significantly associated with firmness and propectin content in the ‘Blue’, ‘Darkslateblue’ and ‘Darkorange’ modules. (DOCX)
