# Supplementary material for: Transcriptome analysis of table grapes (Vitis vinifera L.) identified a gene network module associated with berry firmness
Source: PLoS One. 2020 Aug 17;15(8):e0237526. doi: 10.1371/journal.pone.0237526 (PMC7430731; doi:10.1371/journal.pone.0237526)
Supplement: S1 Table — (DOCX) [file pone.0237526.s001.docx]

**S1 Table** Primers used for RT-qPCR validation.

| Gene ID | Gene name | Sequence | |
| --- | --- | --- | --- |
| LOC104882358 | *VvWAKL8* | Forward (5'-3') | TGGGTTCATCGGTCATCACA |
|  |  | Reverse (5'-3') | CATCTCCGCATCGTTCTTGG |
| LOC100243748 | *VvbHLH36* | Forward (5'-3') | GTCCTCAACACATGCGCACA |
|  |  | Reverse (5'-3') | TGGGAGGAGGGAGTTGAGCT |
| LOC100260158 | *VvEXP* | Forward (5'-3') | AACGTTGGTGGAGCTGGTGA |
|  |  | Reverse (5'-3') | TGCTCCACTGAAGGTCTGGC |
| LOC100265471 | *VvXTH* | Forward (5'-3') | ACGACTATTGCCAGGACCCC |
|  |  | Reverse (5'-3') | TTCTTGGCAAGCTGGACCCA |
| LOC100262510 | *Vvβ-GAL* | Forward (5'-3') | ACCGTCAACCTCCTATGCAA |
|  |  | Reverse (5'-3') | CACGTACCAGTCTCCCTCTC |
| LOC104880619 | *VvPE* | Forward (5'-3') | ATCCCCAGTCCTCAGTGTTG |
|  |  | Reverse (5'-3') | GATTTGACGATGGGGTGAGC |
| LOC100243965 | *VvGATL10* | Forward (5'-3') | TCTCATCTTCCATCCGTCGG |
|  |  | Reverse (5'-3') | AGCTCCCCATCAAACCAGAA |
| LOC100855221 | *VvPG* | Forward (5'-3') | ACGACTATTGCCAGGACCCC |
|  |  | Reverse (5'-3') | TTCTTGGCAAGCTGGACCCA |
| LOC100255011 | *VvPL* | Forward (5'-3') | TGGCGCATCTTGATGGTGGA |
|  |  | Reverse (5'-3') | TGCCTCCACCACCTCAAGAA |
| AF369524 | *VvActin* | Forward (5'-3') | CAAGAGCTGGAAACTGCAAAGA |
|  |  | Reverse (5'-3') | AATGAGAGATGGCTGGAAGAGG |
