# Supplementary material for: Transcriptome analysis of table grapes (Vitis vinifera L.) identified a gene network module associated with berry firmness
Source: PLoS One. 2020 Aug 17;15(8):e0237526. doi: 10.1371/journal.pone.0237526 (PMC7430731; doi:10.1371/journal.pone.0237526)
Supplement: S2 Table — (DOCX) [file pone.0237526.s002.docx]

**S2 Table** Summary statistics for sequence quality control and mapped data of samples

| Samples | Clean reads | Clean bases | GC content (%) | Q30 (%) | Mapped reads (%) | Uniquely mapped reads (%) | Multiple mapped reads (%) |
| --- | --- | --- | --- | --- | --- | --- | --- |
| Red Globe Pre-Veraison 1 | 47638106 | 7117499938 | 47.03 | 94.21 | 87.05 | 84.83 | 2.22 |
| Red Globe Pre-Veraison 2 | 50435996 | 7547923036 | 47.22 | 93.98 | 85.98 | 83.81 | 2.18 |
| Red Globe Pre-Veraison 3 | 41877880 | 6263580206 | 46.85 | 94.14 | 89.05 | 86.89 | 2.16 |
| Muscat Hamburg Pre-Veraison 1 | 44224470 | 6606139664 | 47.2 | 93.87 | 85.28 | 83.24 | 2.04 |
| Muscat Hamburg Pre-Veraison 2 | 44393880 | 6626111604 | 47.1 | 94.09 | 85.91 | 83.88 | 2.03 |
| Muscat Hamburg Pre-Veraison 3 | 45293540 | 6766761990 | 47.51 | 93.99 | 82.8 | 80.75 | 2.05 |
| Red Globe Veraison 1 | 41667058 | 6226464160 | 46.48 | 94.22 | 89.98 | 87.77 | 2.21 |
| Red Globe Veraison 2 | 43955136 | 6557669374 | 46.62 | 92.11 | 87.32 | 85.15 | 2.17 |
| Red Globe Veraison 3 | 46398626 | 6930884542 | 46.89 | 92.43 | 85.7 | 83.45 | 2.25 |
| Muscat Hamburg Veraison 1 | 38608870 | 5756580274 | 47.48 | 93.86 | 83.66 | 81.68 | 1.98 |
| Muscat Hamburg Veraison 2 | 45963786 | 6874607012 | 46.68 | 94.14 | 88.92 | 86.96 | 1.97 |
| Muscat Hamburg Veraison 3 | 45661580 | 6810532702 | 47.06 | 94.22 | 85.75 | 83.78 | 1.97 |
| Red Globe Maturation 1 | 40809728 | 6092299300 | 47.61 | 92.14 | 88.68 | 85.23 | 3.45 |
| Red Globe Maturation 2 | 44395486 | 6624466196 | 48.81 | 94.13 | 83.05 | 79.71 | 3.34 |
| Red Globe Maturation 3 | 58171568 | 8673850092 | 47.78 | 93.56 | 88.81 | 85.22 | 3.58 |
| Muscat Hamburg Maturation 1 | 49454406 | 7389054276 | 47.77 | 93.96 | 81.94 | 79.23 | 2.71 |
| Muscat Hamburg Maturation 2 | 46201670 | 6906185456 | 47.67 | 94.12 | 83.29 | 80.31 | 2.98 |
| Muscat Hamburg Maturation 3 | 55661210 | 8297270824 | 48.17 | 93.98 | 78.66 | 75.84 | 2.82 |
