# Supplementary material for: Transcriptome analysis of table grapes (Vitis vinifera L.) identified a gene network module associated with berry firmness
Source: PLoS One. 2020 Aug 17;15(8):e0237526. doi: 10.1371/journal.pone.0237526 (PMC7430731; doi:10.1371/journal.pone.0237526)
Supplement: S6 Table — (DOCX) [file pone.0237526.s006.docx]

**S6 Table** The firmness and propectin contents significantly associated transcription factors and cell wall metabolism-related genes in ‘Blue’, ‘Darkslateblue’ and ‘Darkorange’ modules.

| **Module Color** | **Transcription factors** | **Cell wall metabolism-related gene** |
| --- | --- | --- |
| Blue | C3H (LOC100265670) | PE (LOC104880619)  PG (LOC100855221)  PL (LOC100255011) |
|  | B3 (LOC100255593) |  |
|  | AP2/ERF-RAV (LOC104880622) |  |
|  | bHLH130 (LOC100855101) |  |
|  | bHLH36 (LOC100243748) |  |
| Darkslateblue | WRKY41 (LOC100253577) | GATL10 (LOC100243965)  XTH (LOC100265471)  WAKL20 (LOC100246196) |
|  | AP2/ERF-ERF (LOC100253072) |  |
|  | MYB3R-1 (LOC100246639) |  |
|  | ERF043 (LOC100249652) |  |
|  | SPL6 (LOC100245772) |  |
|  | NFYB5 (LOC104879763) |  |
|  | ERF053 (LOC100246593) |  |
|  | NAC090 (LOC100263202) |  |
|  | NAC (LOC100252516) |  |
|  | CRF4 (LOC100248494) |  |
|  | Trihelix (LOC100256096) |  |
|  | NAC (LOC100256653） |  |
|  | C2C2-LSD (LOC100265410） |  |
|  | SBP (LOC100243827） |  |
|  | LOB1 (LOC104881937） |  |
|  | RWP-RK (LOC100253057） |  |
|  | LOB15 (LOC100261743） |  |
| Darkorange | NAC (LOC100265122） | WAKL8 (LOC104882358) |
|  |  | β-GAL (LOC100262510) |
